# Increasing access to comprehensive cleft lip and/or palate care in Colombia

**DOI:** 10.15649/cuidarte.4277

**Published:** 2024-12-09

**Authors:** Laura van-der-Werf, Edna Lucia Guerrero-Cortés, Vivian Alexandra Barreto-Gaitán, Andrea Carolina Diaz-Gil, Olga Isabel Sarmiento-Viasus, Diana Catalina Tibaquirá, Álvaro Mauricio Herrera-Cepeda

**Affiliations:** 1 Fundación Operación Sonrisa Colombia, Bogotá, Colombia. E-mail: laura.vanderwerf@operacionsonrisa.org.co Fundación Operación Sonrisa Colombia Bogotá Colombia laura.vanderwerf@operacionsonrisa.org.co; 1 Fundación Operación Sonrisa Colombia, Bogotá, Colombia. E-mail: edna.guerrero@operacionsonrisa.org.co Fundación Operación Sonrisa Colombia Bogotá Colombia edna.guerrero@operacionsonrisa.org.co; 1 Fundación Operación Sonrisa Colombia, Bogotá, Colombia. E-mail: alexandra.barreto@operacionsonrisa.org.co Fundación Operación Sonrisa Colombia Bogotá Colombia alexandra.barreto@operacionsonrisa.org.co; 1 Fundación Operación Sonrisa Colombia, Bogotá, Colombia. E-mail: andrea.diaz@operacionsonrisa.org.co Fundación Operación Sonrisa Colombia Bogotá Colombia andrea.diaz@operacionsonrisa.org.co; 1 Fundación Operación Sonrisa Colombia, Bogotá, Colombia. E-mail: olga.sarmiento@operacionsonrisa.org.co Fundación Operación Sonrisa Colombia Bogotá Colombia olga.sarmiento@operacionsonrisa.org.co; 1 Fundación Operación Sonrisa Colombia, Bogotá, Colombia. E-mail: catalina.tibaquira@operacionsonrisa.org.co Fundación Operación Sonrisa Colombia Bogotá Colombia catalina.tibaquira@operacionsonrisa.org.co; 1 Fundación Operación Sonrisa Colombia, Bogotá, Colombia. E-mail: mauricio.herrera@operacionsonrisa.org.co Fundación Operación Sonrisa Colombia Bogotá Colombia mauricio.herrera@operacionsonrisa.org.co

Cleft lip and palate are the most common congenital cranial malformations. They occur in about 1.7 of every 1000 live births^1^. Although it is likely to be under-reported, it is estimated that the prevalence ofthis condition in Colombia is 3.27 per 10,000 inhabitants^2^.

Comprehensive care for these malformations requires multiple interventions by a specialized multidisciplinary team from pregnancy through adulthood^3^^, ^^6^. Ideally, these services should be organized around the patient's needs and provided by an interdisciplinary team of experts located in the same place who communicate directly with each other to make decisions together^6^. This is in order to positively impact the health outcomes that matter most to patients and their families (Figure 1).

Although insurance coverage in Colombia is practically universal^8^ and the services required by cleft lip and/or palate patients are included in the basic benefit plan financed by the Capitation Payment Unit^9^, access to comprehensive care for people with this condition continues to be limited. Those with lower socioeconomic status and those living in dispersed rural areas are less likely to have access to adequate comprehensive treatment^10^^, ^^11^.

**Figure 1:**
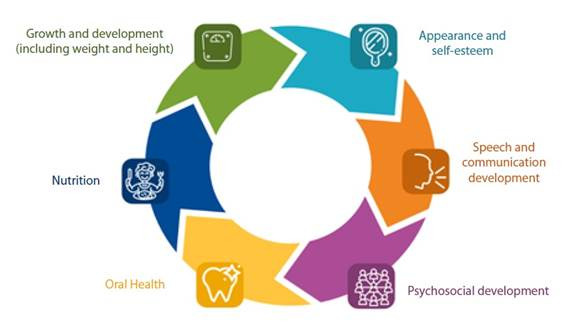
Health outcomes relevant to cleft lip and/or palate patients.

*Source: Adapted from Allori et al.* ^7^

## Prenatal diagnosis and prenatal care

Facial clefts can be seen as early as the twelfth week of pregnancy and are detected by ultrasound between 18 and 22 weeks^12^. However, in Colombia, only 30-36% of cases are detected prenatally^13^^, ^^15^, making early intervention and preparation of parents for their children's needs difficult. The low level of prenatal screening hinders adequate counseling by an interdisciplinary team for parents and families before birth.

## Care in the first months of life

At the time of birth, especially when hospitalization is required, people with these conditions need adequate care to facilitate feeding and promote bonding during the first months of life^16^. Children with cleft palates often experience setbacks in initiating breastfeeding due to difficulties in creating the negative pressure necessary for adequate suckling. This reversal may result in insufficient nutrient intake, which could lead to delays in the newborn's growth and development^17^.

The lack of healthcare professionals experienced in cleft lip and/or palate management to help establish direct breastfeeding or provide alternatives for indirect breastfeeding limits access to comprehensive care for these patients in the first months of life^16^.

## Access to surgical treatment

In Colombia, more than 7.1 million people are more than two hours away from an operating room that can provide essential surgery. The workforce density for providing surgery, obstetrics, and anesthesia is 13.7 per 100,000 population (below the Lancet Commission on Global Surgery recommendation of 20 per 100,000 population). In more impoverished and rural municipalities, there are fewer health workers in these areas, and access to surgical care is more limited^18^.

Access to surgical care for children is even more restricted. For pediatric care, operating rooms require specific equipment, medical devices, and drugs tailored to this population. Over its 30 years of existence, the Fundación Operación Sonrisa has provided devices, medicines, and supplies for pediatric surgery in short-term surgical programs, as many operating rooms in many regions of the country are equipped only for adult care. Biomedical equipment for pediatric monitoring, pediatric anesthesia equipment, and specialized instruments are required, for example, cuffs, oximeters, laryngoscopes, anesthesia masks, and orotracheal tubes, all in pediatric sizes. The availability of these resources and staff training in their use is important to ensure the safety and success of surgeries in children^19^.

The surgical care of patients with cleft lip and/or palate presents additional challenges beyond general pediatric surgery care. Specialized medical devices are required, such as specific sutures for cheilorrhaphy, cheiloplasty, palatorrhaphy and palatoplasty, alveolar bone grafting, and other procedures related to this condition management. In addition, the surgical instruments for these surgeries are highly specialized and are not available in all hospitals in the country^19^.

There are patient safety barriers to consider when managing individuals with these conditions, which may not exist in healthcare settings where these procedures are not commonly performed. These barriers include the World Health Organization's Surgical Safety Checklist^20^, which focuses on cleft lip and/or palate patients^21^; measures to prevent airway obstruction caused by gauze in the mouth, including visible warnings in the operating room; and completion of instrument, sponge, and needle counts before aspirating secretions and removing the orotracheal tube^21^.

Anesthesiologists may encounter difficulties when caring for pediatric patients with this condition due to differences in facial anatomical structure, which can complicate safe airway management and increase the risk of anesthetic complications^22^. In the case of nurses, although basic training includes pediatric care, many nurses may not have sufficient experience with this population. For example, peripheral venous access can be difficult to achieve due to the particularities of the anatomy of the pediatric population^23^. In addition, due to the rarity of this condition, many nurses may not be specifically trained to manage patients with this condition in the perioperative period.

Surgical technologists require specific training and sufficient exposure to various procedures to know and handle the specialized instruments used in cleft lip and palate surgeries^24^^, ^^27^.

As for the availability of surgeons, specialized training is required to perform procedures such as cheilorrhaphy, cheiloplasty, palatorrhaphy and palatoplasty, and alveolar bone grafting. Plastic surgeons may not necessarily have sufficient exposure to these cases during residency, requiring further training to perform these procedures. Not having specialized training for the specifics of these procedures may result in an increase in surgical complications^28^. The limited availability of surgeons trained in facial cleft surgery and their concentration in major cities pose a barrier to timely access to cleft lip and/or palate surgeries.

## Access to speech therapy

Children born with cleft palate may encounter challenges in communicating effectively. As such, evaluating and treating communication and speech disorders associated with cleft palate are critical aspects of comprehensive treatment^29^. In order to counsel, evaluate, and treat these patients, it is necessary to develop special skills through auditory exposure to the speech alterations characteristic of this condition. Acquiring these skills is related to sufficient exposure to cases by professionals, so it is common for speech-language pathologists to report that they do not have the necessary training or experience to manage these patients.

The limited availability of professionals trained in providing speech therapy for patients with these conditions and their concentration in the country's major cities represents a significant barrier to comprehensive care for individuals with cleft lip and/or palate in Colombia.

## Access to dental care

Orthodontics is an essential part of the comprehensive management of cleft lip and/or palate^7^. In addition, cleft lip and/or palate patients have a higher prevalence of dental caries and periodontal disease^30^^, ^^33^, making access to dental care particularly important for them^34^. Access to dental care for the general population in Colombia is limited and maybe even more difficult for patients with this condition due to the limited experience of dental professionals with this population and the fear of palatal dehiscence by both professionals and caregivers.

## Access to multidisciplinary care

Cleft lip and/or palate care should be provided by a specialized team that works together in a coordinated manner to address all of the patient's needs. However, because of the way insurers in Colombia contract with different healthcare providers, the care of patients with the disease is often segmented, with each specialty working in isolation. This further exacerbates the difficulties in accessing care and the costs associated with transportation as they have to go to different places to receive care^5^.

## Initiatives of the Fundación Operación Sonrisa Colombia

Throughout its 30-year history, Fundación Operación Sonrisa Colombia has developed strategies to improve access to comprehensive treatment for people with cleft lip and/or palate. Short-term surgical programs are a strategy to bring specialized cleft lip and/or palate care to parts of the country where it is unavailable. During these programs, a specialized team travels to provide multi-specialty care and, for patients requiring it, surgical care.

Fundación Operación Sonrisa Colombia's surgical programs have evolved from a vertical approach -targeting specific health conditions and offering services in parallel but not necessarily integrated into the local health system-to a diagonal approach. This combination consists of finding a synergy between the immediate advantages of vertical inputs (such as surgical missions) and the long-term benefits of horizontal objectives, which include long-term investments in health infrastructure and the expansion of publicly funded health systems^12^. As a result, the Foundation's programs have become generators for building local capacities by training healthcare personnel and strengthening infrastructure for health service delivery.

The Foundation has developed strategies to improve access to multidisciplinary care, adapted to the context of different regions of the country. These strategies include multidisciplinary care at the Multidisciplinary Care Center in Bogota, multidisciplinary care in community settings and the homes of patients and their families in La Guajira, and the formation of inter-institutional alliances for the continuous multidisciplinary care of patients with this condition in different regions of the country.

In conclusion, ensuring access to comprehensive cleft lip and palate care in Colombia requires overcoming multiple challenges, from prenatal detection to the provision of appropriate surgical and rehabilitation services. Despite the almost universal coverage of the health insurance system, geographic, economic, and infrastructure barriers prevent many patients from accessing the care they need.

Fundación Operación Sonrisa has worked to overcome these barriers through its short-term surgical programs and efforts to strengthen local capacity. By evolving from a vertical to a diagonal approach, the Foundation not only provides direct interventions, but also invests in building human talent and improving health infrastructure to create lasting impact in the communities it serves.

In order to move forward, it is essential to ensure that there are adequately trained professionals to manage the complexity of care for people with this condition. Only a coordinated and sustainable approach can ensure comprehensive, quality care for all patients, regardless of location or socioeconomic status. Continued collaboration among institutions, governments, and nongovernmental organizations will be essential to overcome these challenges and improve health outcomes for people with this condition in Colombia.
